# Self-reported gait unsteadiness in mildly impaired neurological patients: an objective assessment through statistical gait analysis

**DOI:** 10.1186/1743-0003-9-64

**Published:** 2012-08-29

**Authors:** Maria Grazia Benedetti, Valentina Agostini, Marco Knaflitz, Verusca Gasparroni, Marco Boschi, Roberto Piperno

**Affiliations:** 1 Physical Medicine and Rehabilitation Unit, Istituto Ortopedico Rizzoli, Bologna, Italy; 2 Dipartimento di Elettronica e Telecomunicazioni, Politecnico di Torino, Torino, Italy; 3 Movement Analysis Laboratory, Istituto Ortopedico Rizzoli, University of Bologna, Bologna, Italy; 4 Rehabilitation Division, Maggiore Hospital, Bologna, Italy

## Abstract

**Background:**

Self-reported gait unsteadiness is often a problem in neurological patients without any clinical evidence of ataxia, because it leads to reduced activity and limitations in function. However, in the literature there are only a few papers that address this disorder. The aim of this study is to identify objectively subclinical abnormal gait strategies in these patients.

**Methods:**

Eleven patients affected by self-reported unsteadiness during gait (4 TBI and 7 MS) and ten healthy subjects underwent gait analysis while walking back and forth on a 15-m long corridor. Time-distance parameters, ankle sagittal motion, and muscular activity during gait were acquired by a wearable gait analysis system (Step32, DemItalia, Italy) on a high number of successive strides in the same walk and statistically processed. Both self-selected gait speed and high speed were tested under relatively unconstrained conditions. Non-parametric statistical analysis (Mann–Whitney, Wilcoxon tests) was carried out on the means of the data of the two examined groups.

**Results:**

The main findings, with data adjusted for velocity of progression, show that increased double support and reduced velocity of progression are the main parameters to discriminate patients with self-reported unsteadiness from healthy controls. Muscular intervals of activation showed a significant increase in the activity duration of the Rectus Femoris and Tibialis Anterior in patients with respect to the control group at high speed.

**Conclusions:**

Patients with a subjective sensation of instability, not clinically documented, walk with altered strategies, especially at high gait speed. This is thought to depend on the mechanisms of postural control and coordination. The gait anomalies detected might explain the symptoms reported by the patients and allow for a more focused treatment design. The wearable gait analysis system used for long distance statistical walking assessment was able to detect subtle differences in functional performance monitoring, otherwise not detectable by common clinical examinations.

## Background

Gait and balance disorders cause severe impairments for patients affected by cerebellar ataxia, who are at risk of accidental falls and disability in daily life activities [1,2]. Several studies in the literature describe ataxic gait anomalies. Specifically time-distance parameters were found to be altered in terms of decreased velocity both for decreased step length and cadence, increased double-support phase [2-7], increased step width [2,4,5,8,9], and irregular timing and amplitude of steps [6,9]. Increased thigh and shank muscle activity with co-contraction [5,7] and decomposition of joint movements in the legs [3,7,8] were also reported. Gait abnormalities were commonly interpreted as compensatory reactions for instability in walking [5-7,9]. However a dysfunctional gait control in terms of activity between agonist and antagonist muscles was also supposed [5] based on the finding of excessive muscle activity during walking, particularly at the ankle where co-contraction of the tibialis anterior and gastrocnemius-soleus couple was identified. Further studies found a strict interrelation between balance deficits and cerebellar gait ataxia [4,8]^.^

Most of the literature concerns patients affected by diagnosed ataxia. Patients with early Multiple Sclerosis (MS) and patients with good recovery after a Traumatic Brain Injury (TBI) (i.e., the patient has resumed most normal activities) also often complain of gait and balance symptoms. However, the diagnosis of ataxia or postural imbalance is often not substantiated in these patients, and subjective unsteadiness during gait is the only symptom patients complain about. Actually this symptom can be considered as emergent in multifocally brain-damaged patients, regardless of putative prevailing involvement of the proprioceptive, vestibular or cerebellar networks. Little is known about the gait patterns of these mildly neurologically impaired patients. Subjective complaints of impaired balance are particularly troublesome with as many as 30% of TBI patients complaining of these problems even without clear neurological deficits [10,11] , and as many as 24% of MS patients with no ADL difficulty [12]. Therefore we have focused our attention on a group of patients with no functional limitations in normal daily activities that are frequently addressed to our rehabilitation facilities for subjective unsteadiness and/or dizziness while walking. This group of patients can be classified as highly-functioning because they can walk independently at home and in moderate community activities, can accept uneven terrain and can negotiate a crowded shopping center (“community walker”) [13].

An objective assessment of self-reported symptoms is needed to refine the diagnostics and to prescribe an appropriate rehabilitation training program. Indeed, it is not trivial to define measurable gait parameters that correlate to the sensation of instability in these patients with almost normal Romberg test results.

The aim of this study was to measure gait variables in patients with a self-reported sensation of unsteadiness during gait. This symptom is not clinically substantiated by common neurological tests, but reported by patients as a discomfort limiting their participation in social and leisure activities. The hypothesis of the present work is that self-reported instability might correspond to underlying abnormal gait strategies which are detectable only by means of appropriate instruments during walking. For this purpose, a multichannel recording system for statistical gait analysis on a high number of consecutive strides was used in an unconstrained experimental setting. Both self-selected and high gait speed were explored to study the context of symptoms onset better.

## Methods

A sample of 11 subjects were recruited from the patients referred to our rehabilitation facilities. Seven patients were affected by Multiple Sclerosis (relapsing-remittent form; EDSS score <4) and four patients had a severe TBI (initial GCS score < =8) with good recovery according to the Glasgow Outcome Scale (GOS). Eight were men and three were women with a mean age of 33.5 ± 10.4 years, a mean height of 171,4 ± 11,5 cm, and a mean weight of 69,6 ± 9,9. Ten healthy subjects, five men and five women, mean age 28,5 ± 3 years, mean height of 170,3 ± 6,2 cm, and a mean weight of 64,7 ± 9, with a normal lifestyle were enrolled among clinical postgraduate residents as control group. All the subjects signed an informed consent form to participate in the study which was approved by our Institutional Scientific Board.

All the patients were living in the community with no functional limitations and no need for supervision in daily living activities, except for a subjective, self-reported, unsteadiness while walking. Cognitive or behavioral problems potentially interfering with motor activities or with the participation to the study were exclusion criteria. All the patients have been clinically examined by the same senior neurologist (RP). They had no clinically detectable signs of cerebellar involvement in terms of limb and/or gait ataxic features, no direct cerebellar lesions detected by neuroimaging, and the Romberg’s test was held for at lest 30 sec. both in open and closed eyes condition. All the patients had normal musculoskeletal examination and absence of spasticity and paresis. Other mild neurological signs (increased deep tendon reflexes, Babinski’s reflex, reduced pallestesia, mood disorders), when present, were assumed not to affect the gait.

Gait analysis data were acquired by a multichannel recording system for statistical gait analysis (Step32, DemItalia, Italy). The following parameters were considered:

· Time distance parameters: gait cycle duration (seconds), cadence (strides/min), single and double support phases duration expressed as a percent of the gait cycle (GC), swing phase duration (percent of the GC).

· Sub-phases of stance: Time of heel contact (H), Time of flat foot contact (heel and forefoot contact - F) and Time of forefoot contact (from heel rise to toe off - P) (percent of the stance phase).

· Ankle kinematics in the sagittal plane: angles of maximum dorsiflexion in stance and maximum plantarflexion during swing (degrees).

· Muscular activity during the gait cycle expressed as percent of GC.

**Figure 1:**
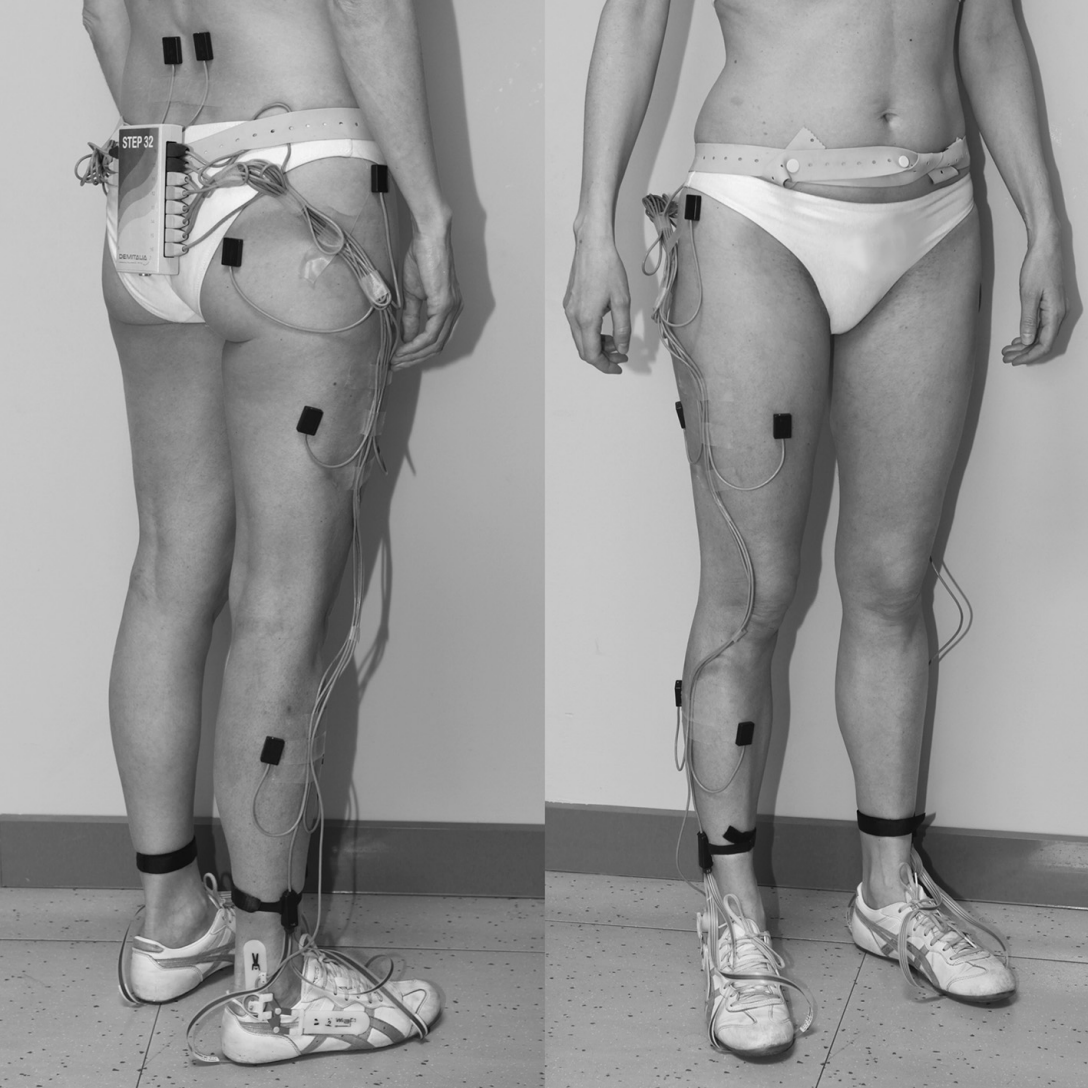
A subject instrumented with the STEP 32 system.

Each subject was instrumented with foot-switches, ankle goniometers, and surface electromyographic (SEMG) probes. Three foot-switches (size: 10 mm × 10 mm ×0.5 mm; activation force = 3 N) were attached beneath the heel, and the first and fifth metatarsal heads of each foot to obtain the foot-floor contact phases. An electrogoniometer (accuracy: 0.5 deg) was attached to the lateral side of the limb for measuring the ankle joint angles in the sagittal plane. SEMG probes were attached on the skin over the Tibialis Anterior (TA), Gastrocnemius Lateralis (GAS), Rectus Femoris (RF), Lateral Hamstrings (LH), Gluteus Medius (GME), and Gluteus Maximum (GMAX). Furthermore, probes were attached to the skin over the Erector Spinae, bilaterally (RES and LES). An example of subject’s instrumentation is shown in Figure 1. Probes were positioned according to the guidelines suggested by Delagi et al. [14]. The time-distance parameters obtained from the left and right gait cycles of each subject were averaged to calculate the subject mean data.

SEMG signals were high-pass filtered (FIR filter, 100 taps, cut-off frequency equal to 20 Hz) to attenuate movement artifacts and then processed by a double-threshold statistical detector that gives, in a user-independent way, the muscle activation intervals [15,16].

To obtain an ensemble average of the results it is convenient to study gait cycles relative to strides recorded during a walk along a straight walkway. The subject is asked to walk back and forth along the straight path: he/she is instructed to walk from the starting point until the end of the straight path, turn 180° and walk back to the initial point, turn again 180°, start walking again along the path and so on.

During acceleration, deceleration, and changes in direction the strides are different from those of steady state walking. Therefore, strides involving deceleration, reversing and acceleration are removed automatically by the Step32 system, using a multivariate statistical filter that detects and eliminates those “outliers” [16]. All the subjects were asked to walk back and forth at their self-selected speed and at the maximum possible speed, wearing their comfortable shoes, on a 15-m long corridor.

Statistical analyses were carried out on the data obtained. All continuous data were expressed in terms of the mean and the standard deviation of the mean. The Mann–Whitney test was performed on unpaired data (control vs. patient group at self-selected and high speed), whereas the Wilcoxon test was carried out on paired data (control group at self-selected speed vs. high speed, patient group at self-selected vs. high speed). Spearman’s Correlation was used to determine which gait parameters might depend on the velocity within the two groups. A multivariate analysis was finally carried out on parameters that were dependent on the velocity by the General Linear Model (GLM) with controls/patients as the fixed effect and velocity as a covariate. The multivariate Logistic Regression with Wald’s backward method was performed to confirm parameters differentiating the two groups. For all tests, p < 0.05 was considered significant. Statistical analyses were carried out using the Statistical Package for the Social Sciences (SPSS) software version 15.0 (SPSS Inc., Chicago, USA).

## Results

### Time-distance parameters

The mean number of gait cycles analyzed after removing the outliers was 65.5 ± 27.8 for patients at self-selected speed and 63 ± 27 at high speed, while controls walked an average number of 57.3 ±19.7 gait cycles at self-selected speed and 51.4 ±22.1 gait cycles at high speed. The speed of progression during walking at self-selected speed was 0.94 ± 0.18 m/s for patients and 1.44 ± 0.32 m/s for controls (Table 1). When walking at high speed, the value was 1.24 ± 0.18 m/s for patients and 1.79 ± 0.22 m/s for controls. Patients showed a decreased walking speed with respect to controls, both at self-selected (p < 0.005) and at high speed (p = 0.023). A significant increase in the double-support phase at self-selected speed (p = 0.004) was found in patients with respect to controls.

**Table 1:** Time- distance parameters

		**Self selected speed**			**High speed**		
		***Mean***	***SD***	***Mann Whitney test***	***Mean***	***SD***	***Mann Whitney test***
				***p***			***p***
N. Gait cycle	*Patients*	65,5	*27,8*	*ns*	63,0	*27,0*	*ns*
	*Controls*	57,3	*19,7*		51,4	*22,1*	
Cycle duration (s)	*Patients*	1,2	*0,2*	*ns*	1,0	*0,2*	*ns*
	*Controls*	1,1	*0,1*		0,9	*0,1*	
Cadence (str/m)	*Patients*	48,6	*6,8*	*ns*	61,1	*10,2*	*=0,05*
	*Controls*	53,9	*2,5*		69,0	*6,7*	
Velocity (m/s)	*Patients*	0,9	*0,2*	*<0,005*	1,2	*0,2*	*=0,023*
	*Controls*	1,4	*0,3*		1,8	*0,2*	
Swing phase (%GC)	*Patients*	35,2	*7,5*	*ns*	34,4	*11,9*	*ns*
	*Controls*	39,7	*2,9*		40,7	*4,5*	
Single support (%GC)	*Patients*	35,8	*5,0*	*ns*	35,9	*8,4*	*ns*
	*Controls*	38,8	*4,3*		42,1	*5,5*	
Double support (%GC)	*Patients*	29,2	*6,3*	*0,004*	28,5	*9,5*	*=0,019*
	*Controls*	20,7	*4,3*		19,2	*6,2*	

A different distribution of stance sub-phase duration was also evident (Table 2). In particular, F increased (p = 0.01), the heel-rise appeared delayed and the P phase was shortened (p = 0.014) at self selected speed.

**Table 2:** Stance sub-phases

		**Selef selected speed**			**High speed**		
		***Mean***	***SD***	***Mann Whitney test***	***Mean***	***SD***	***Mann Whitney test***
				***p***			***p***
Time of heel contact H (% stance)	*Patients*	5,15	2	ns	5,67	5,6	ns
	*Controls*	4,9	1,43		5,34	2,01	
Time of flat foot contact F (% stance)	*Patients*	41,45	8,82	=0,01	35,56	8,19	=0,04
	*Controls*	30,83	6,83		28,23	9,38	
Time of forefoot contact P (% of stance)	*Patients*	19,2	5,21	=0,014	26,69	9,75	ns
	*Controls*	24,43	4,69		23,71	9,62	

At high walking speed, cadence was reduced in patients with respect to controls (p = 0.05). Double-support phase was unchanged in patients, but decreased in controls (p = 0.019) (Table 1). The duration of the F sub-phase decreased, but still remained longer with respect to controls (p = 0.04) (Table 2). The P sub-phase increased in patients, even if it was not statistically different from that of controls.

### Ankle Joint Angles

**Figure 2:**
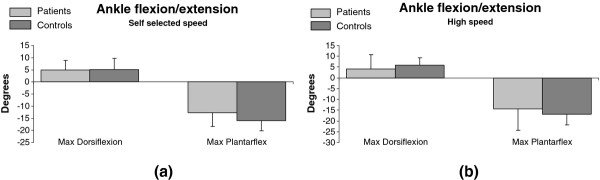
**Maximum angle of ankle plantar-flexion and dorsi-flexion (degrees): mean value and standard deviation.** Light grey bars correspond to patients, dark grey bars to controls. Self-selected speed (**a**) and high speed (**b**) values are reported.

No significant variations were found with respect to the maximum angles of dorsi and plantar-flexion during the gait cycle in both speed conditions (Figure 2a and 2b).

### Muscular activity

**Figure 3:**
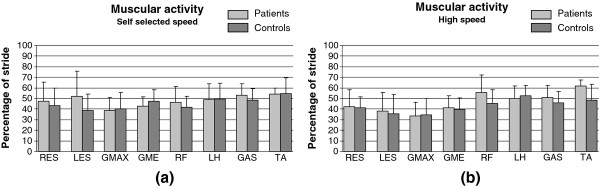
**Muscular activity means value and standard deviations. The value reported for each muscle corresponds to the percentage of stride in which the muscle is active.** The muscles explored were Right and Left Erector Spinae (RES, LES), Gluteus Maximum (G MAX), Gluteus Medius (GME), Rectus Femoris (RF), Lateral Hamstrings (LH), Lateral Gastrocnemius (GAS) and Tibialis Anterior (TA). Light grey bars correspond to patients, dark grey bars to controls. Self-selected speed (**a**) and high speed (**b**) values are reported. A significant difference was found for RF ((p < 0.02) and TA (p < 0.008) when compared within the patients group at different speed.

Muscular intervals of activation were not different in the two groups both at self-selected and high speed (Figure 3a and 3b).

### Additional statistical analyses

No differences were found between the two groups in terms of age, weight and height. When the gait patterns at self-selected speed and at high speed within the two groups were compared, the expected increment of cadence was evident only in the control group (p < 0.002). No other differences were found in the control group switching from a self-selected speed to high speed of progression.

In the patient’s group, the F phase was reduced (p < 0.004), although it was still longer than that of the control group, whereas the P sub-phase increased (p < 0.03). Muscular activity increased for RF (p < 0.02) and TA (p < 0.008).

Univariate analysis showed that some parameters were correlated with velocity: they were cadence (p < 0.0005), single support (p < 0.0005), double support (p = 0.09), maximum angle of plantarflexion during swing (p = 0.001), and duration of activation of RES (p = 0.031) and TA (p = 0.043). To test whether, at a similar speed, there was a difference between patients and controls; the GLM was applied to the variables depending on velocity. Only the double support was found to be different between groups (p = 0.005, partial eta squared = 0.196). This result was also confirmed with the logistic regression with backward Wald method (OR = 1.172, 95% Interval of Confidence 1.031-1.332, p = 0.015). With this method velocity was also different between groups (OR = 0,042, Interval of Confidence 0.002-0.736, p = 0.03).

## Discussion

The aim of this study was to measure gait variables in patients affected by MS and TBI with a self-reported sensation of unsteadiness not clinically detectable. The hypothesis was that self-reported instability might correspond to underlying subclinical abnormal gait strategies which might justify the discomfort reported by the patient. Gait analysis both at self-selected and high velocity of progression was carried out to detect the instability claimed by patients. To better explore the context of symptom onset in an unconstrained experimental setting [17], an advanced wearable technology for gait analysis was used.

Results confirm the hypothesis that gait anomalies are actually detectable in patients without clinical signs of ataxia, but with self-reported sensation of unsteadiness during gait. The main findings, with data adjusted for velocity of progression, show that increased double support and reduced velocity of progression are the main parameters to discriminate patients from controls. When looking at parameters describing the gait pattern of patients with respect to controls, a cadence decrease and a prolonged flat foot phase support during stance were present, both at self-selected and high walking speed. Moreover, at high speed, patients walked with a prolonged activity of TA and RF muscles with respect to self-selected speed, which was mainly evident at the transition between stance and swing phase, corresponding to the second phase of double support. Studies on healthy subjects show that RF activation occurs with the aim of starting the swing phase by flexing the hip and simultaneously preventing excessive knee flexion. Increasing its activity in this phase has been shown to be related to increasing of speed [18]. However, whereas in the present study TA activity was strictly related to velocity, the activity of RF does not depend on velocity. This might support studies where augmented activity of thigh muscles was reported during stance in ataxic patients [5] as a compensatory reaction to instability in walking [6].

These gait abnormalities are very similar to those reported in patients with an established diagnosis of ataxia and, in general, in most neurological conditions where balance problems are emerging features and the slowness of walking is the compensation [7]. A similar “cautious” gait has already been reported both for TBI [19] and MS patients [20,21]. Nevertheless, recent studies [4] indicate that the cerebellar ataxic gait is influenced by both balance-related impairments and deficits related to dynamic limb control. More specific abnormalities described in ataxic patients, such as stiffening the ankle joint as a strategy of decomposition [8], were not present in the patients with the subjective unsteadiness included in the present study. It is possible that their condition represents a mild expression of ataxia, and only subtle gait anomalies are present with respect to patients with a diagnosed ataxia.

Although a cerebellar damage is the prominent cause of gait and balance ataxia, also impairments in integrating cerebellar input/output or proprioception, without any direct cerebellar lesion, can cause gait and balance incoordination. Disconnection must be therefore considered among the causes of ataxic gait. Polytopic disconnecting lesions in normal-looking white matter can be thought to cause ataxia. Both MS and TBI have aspects of diffuse axonal injury (DAI) that can sustain cognitive, mood and behavioral symptoms as they can impair connection in sensory and/or cerebellar systems, also when no direct cerebellar damage can be revealed. However, DAI seems to affect differently these networks in MS and TBI: in most people with MS, gait ataxia and balance dyscontrol are probably due primarily to slowed somatosensory conduction and impaired integration of proprioception [22], whereas after severe TBI they can arise primarily from involvement of the cerebello-cortical connection, with prevailing damage in the superior cerebellar peduncles [23,24]. DAI can occur in MS [25] and in TBI even in the absence of clinical disability, causing widespread tissue damage and disconnection in normal-looking white matter. Some disconnection in the sensory and/or cerebellar system can therefore exist already when dysfunctional patterns of coordination are not yet clearly discernible: in these cases the only related symptom might be a subjective complaint of unsteadiness during gait and attention-demanding balance tasks in daily activities. Because of the common putative background, we can refer to these patients as affected by “subclinical” ataxia: disentangling “subclinical” and “mild” ataxia, at least in most cases, seems to be only a matter of structured functional assessment and/or paraclinical tools (movement analysis techniques). For instance, in MS patients, balance disorders can be detected already when the Romberg test is still negative, with changes mainly affecting the control strategies rather than the movement parameters [26].

The similarity of gait abnormalities observed in high-functioning subjects with distributed CNS damage, regardless of proprioceptive or cortico-cerebellar prevailing involvement, can strengthen the hypothesis that, in the absence of localized cerebellar lesions, the DAI-related gait anomalies are mostly expression of disconnection in widely distributed systems, probably influenced by compromised fine-tuned balance reactions or by higher-order [27] gait disturbances. This can explain the discrepancy in biomechanical findings when specific gait parameters and muscular behavior are instrumentally explored, with respect to clinical neurological examination.

In conclusion, although the study was conducted on a small sample of patients, its original contribution was to provide instrumental evidence that subtle gait abnormalities are present in patients whose complaints of persistent walking unsteadiness may not be justified by the outcome of the routine neurological examination. This can be of interest for clinicians to plan possible intervention strategies.

## Competing interests

The authors declare that they have no competing interests.

## Authors' contributions

MGB, MK and RB contributed to the conception and design of the study, interpretation of data, and supervising the manuscript critically for intellectual content and technical and medical precision. VG, VA and MB helped in data collection and analysis, VG in drafting the manuscript, and VA was involved in statistical analysis. All authors read and approved the final manuscript.

## References

[B1] Bastian AJ Mechanisms of ataxia Phys Ther 1997 77 672 675 9184691 10.1093/ptj/77.6.672

[B2] Dietz V Neurophysiology of gait disorders: present and future applications Electroenc Clin Neuroph 1997 103 333 355 10.1016/S0013-4694(97)00047-7 9305281

[B3] Earhart GM Bastian AJ Selection and coordination of human locomotor forms following cerebellar damage J Neurophysiol 2001 85 759 769 11160510 10.1152/jn.2001.85.2.759

[B4] Ilg W Golla H Their P Giese MA Specific influences of cerebellar dysfunctions on gait Brain 2007 130 786 798 10.1093/brain/awl376 17287287

[B5] Mitoma H Haayashi R Yanagisawa N Tsukagoshi H Characteristic of parkinsonian and ataxic gaits: a study using surface electromyograms, angular displacements and floor reaction forces J Neurol Sci 2000 174 22 39 10.1016/S0022-510X(99)00329-9 10704977

[B6] Palliyath S Hallett M Thomas SL Lebiedowska MK Gait in Patients with cerebellar ataxia Mov Dis 1998 6 958 964 10.1002/mds.870130616 9827622

[B7] Hallett M Cerebellar ataxic gait. Adv Neurol 2001 87 155 63 11347218

[B8] Morton SM Bastian AJ Relative contributions of balance and voluntary leg-coordination deficits to cerebellar gait ataxia J Neurophysiol 2003 89 1844 1856 12612041 10.1152/jn.00787.2002

[B9] Ebersbach G Sojer M Valldeoroiola F Wissel J Muller J Tolosa E Poewe W Comparative analysis of gait in Parkinson’s disease, cerebellar ataxia and subcortical arteriosclerotic encephalopathy Brain 1999 122-7 1349 1355 10388800 10.1093/brain/122.7.1349

[B10] Geurts AC Ribbers GM Knoop JA van Limbeek J Identification of static and dynamic postural instability following traumatic brain injury Arch Phys Med Rehabil 1996 77 7 639 44 10.1016/S0003-9993(96)90001-5 8669988

[B11] Basford JR Chou LS Kaufman KR Brey RH Walker A Malec JF Moessner AM Brown AW An assessment of gait and balance deficits after traumatic brain injury Arch Phys Med Rehabil 2003 84 3 343 9 10.1053/apmr.2003.50034 12638101

[B12] Kraft GH Freal JE Coryell JK Disability, disease duration, and rehabilitation service needs in multiple sclerosis: patient perspectives Arch Phys Med Rehabil 1986 Mar 67 3 164 8 10.1016/0003-9993(86)90060-2 3954578

[B13] Perry J Garrett M Gronley JK Mulroy SJ Classification of Walking Handicap in the Stroke Population Stroke 1995 26 982 989 10.1161/01.STR.26.6.982 7762050

[B14] Delagi EF Perotto AD Anatomic Guide for The Electromyographer: trunk and limbs 1994 Springfield, Ill., USA: Charles C. Thomas Publisher LTD

[B15] Bonato P D’Alessio T Knaflitz M A statistical method for the measurement of muscle activation intervals from surface myoelectric signal during gait IEEE Trans Biomed Eng 1998 45 287 299 10.1109/10.661154 9509745

[B16] Agostini V Knaflitz M Rajendra Acharya U, Filippo M, Toshiyo T, Subbaram Naidu D, Jasjit SS Statistical Gait Analysis, Chapter 7 Distributed Diagnosis and Home Healthcare 2012 California, (USA): American Scientific Publishers, Stevenson Ranch, California(USA) 99 121 Volume 2

[B17] Zijsltra W Aminian K Mobility assessment in older people; new possibilities and challenges European Journal of Ageing 2007 4 3 12 10.1007/s10433-007-0041-9 PMC5546360 28794767

[B18] Schmitz A Silder A Heidersheit B Mahoney J Thelen DG Differences in lower extremity muscular activation during walking between healthy older and young adults J Electromyogr Kinesiol 2009 19 1085 1091 10.1016/j.jelekin.2008.10.008 19081734 PMC3689417

[B19] McFadyen BJ Swaine B Dumas D Durand A McFadyen BJ Swaine B Dumas D Durand A Residual effects of a traumatic brain injury on locomotor capacity: a first study of spatiotemporal patterns during unobstructed and obstructed walking J Head Trauma Rehabil 2003 18 6 512 25 10.1097/00001199-200311000-00005 14707881

[B20] Benecke R Conrad B Bauer JJ, Poser CM, Ritter W Evaluation of motor deficits in patients suffering from Multiple Sclerosis Progress on Multiple Sclerosis 1980 Berlin, Germany: Springer-Verlag 589 595

[B21] Benedetti MG Piperno R Simoncini L Bonato P Tonini A Giannini S Gait abnormalities in minimally impaired multiple sclerosis patients Mult Scler 1999 5 363 368 10516781 10.1177/135245859900500510

[B22] Cameron MH Lord S Postural Control in Multiple Sclerosis: Implications for Fall Prevention Curr Neurol Neurosci Rep 2010 10 407 412 10.1007/s11910-010-0128-0 20567946

[B23] Haggard P Miall RC Wade D Fowler S Richardson A Anslow P Stein J Damage to cerebellocortical pathways after closed head injury: a behavioural and magnetic resonance imaging study J Neurol Neurosurg Psychiatry 1995 58 433 438 10.1136/jnnp.58.4.433 7738549 PMC1073428

[B24] Hong JH Kim OL Kim SH Lee ML Jang H Cerebellar peduncle injury in patients with ataxia following diffuse axonal injury Brain Res Bull 2009 80 30 35 10.1016/j.brainresbull.2009.05.021 19505539

[B25] De Stefano N Narayanan S Simon J Francis SJ Smith S Mortilla M Tartaglia MC Bartolozzi ML Guidi L Federico A Douglas L Arnold DL Diffuse axonal and tissue injury in patients with multiple sclerosis with low cerebral lesion load and no disability Arch Neurol 2002 59 10 1565 71 10.1001/archneur.59.10.1565 12374493

[B26] Corradini ML Fioretti S Leo T Piperno R Early recognition of postural disorders in multiple sclerosis through movement analysis: a modeling study IEEE Trans Biomed Eng 1997 44 11 1029 38 10.1109/10.641330 9353982

[B27] Nutt JG Freund HJ, Jeannerod M, Hallet M Higher-order disorders of gait Higher-order motor disorders: from neuroanatomy and neurobiology to clinical neurology 2005 Oxford, UK: Oxford University Press 237 248

